# Laparoscopic treatment of a large cystic adenomyosis of the uterus: A case report

**DOI:** 10.1016/j.ijscr.2020.04.084

**Published:** 2020-05-12

**Authors:** Congqing Li, Youjiang Xu, Lin Cong

**Affiliations:** a Department of Obstetrics and Gynecology, The First Affiliated Hospital of Anhui Medical University, no. 218 Jixi Road, Hefei, 230022, China; b Department of Obstetrics and Gynecology, The Second Hospital of Anhui Medical University, no. 678 Furong Road, Hefei, Anhui, 230601, China

**Keywords:** Adenomyosis, Cystic adenomyosis, Endometriosis, Laparoscopy

## Abstract

**Introduction:**

We describe a patient with a large cystic adenomyosis of the uterus who was treated by a laparoscopic approach.

**Case presentation:**

A 38-year-old woman presented with dysmenorrhoea for 4 months. A 104 mm × 55 mm × 60 mm cystic mass was detected on the right side of the posterior wall of the uterus by ultrasound. Laparoscopy was performed, during which a bumpy mass was discovered protruding from the surface of the uterine myometrium. Both ovaries were normal. When the wall of the cystic mass was opened, chocolate-colored fluid was visible. The cyst was completely removed, and postoperative histopathology confirmed a cystic adenomyosis. One month later, the uterus was found to be normal on ultrasound.

**Discussion:**

Endometriosis is a common disease in women and is divided into ovarian endometrioid cysts, superficial endometriosis, deep infiltrating endometriosis and other special types according to pathological characteristics. As a special type of endometriosis, the incidence of cystic adenomyosis is low, and its specific incidence is unknown. Cystic adenomyosis is clinically characterized by the appearance of one or more cystic cavities between the walls of the uterine muscles.

**Conclusion:**

Laparoscopic surgery is an effective method for the diagnosis and treatment of large cystic masses in the uterine myometrium.

## Highlights

• Cystic adenomyosis is rarely reported in the medical literature.
• It is easy for clinicians to misdiagnose or miss the diagnosis because of its low incidence, rare clinical and atypical clinical features.
• In case of large cystic adenomyoma, the surgeon can diagnose and treate the patient with laparoscopic.

## 1 Introduction

Adenomyosis is a disease caused by endometrial glands and the stromal invasion of the uterine myometrium. Usually, patients suffer from symptoms including irregular menstruation and progressive dysmenorrhoea [1]. Adenomyoma shows limited growth and forms nodules or clumps, similar to muscle wall myomas [2]. Cystic adenomas are rare. Giovanni reported a case of a giant cystic adenomyoma with symptoms and signs suggesting uterine malformations that was diagnosed and treated by hysteroscopy [3]. Patients with cystic adenomyoma usually complain of non-specific symptoms, such as abnormal uterine bleeding, chronic pelvic pain and dysmenorrhoea, which are often resistant to therapies with analgesics or cyclic oral contraceptives. Patients with mild symptoms, fertility requirements and patients near menopause could choose conservative treatment with drugs, such as gestrinone, GnRh-α or levonorgestrel intrauterine sustained release system (LNG-IUS). However, for patients with obvious symptoms and larger cystic adenomas, surgery may be necessary. Here, we report a rare case of a large cystic adenomyoma in which we diagnosed and treated the patient with laparoscopy. This study is reported according to the surgical Case REport (SCARE) criteria [4].

## 2 Case presentation

**Fig. 1:**
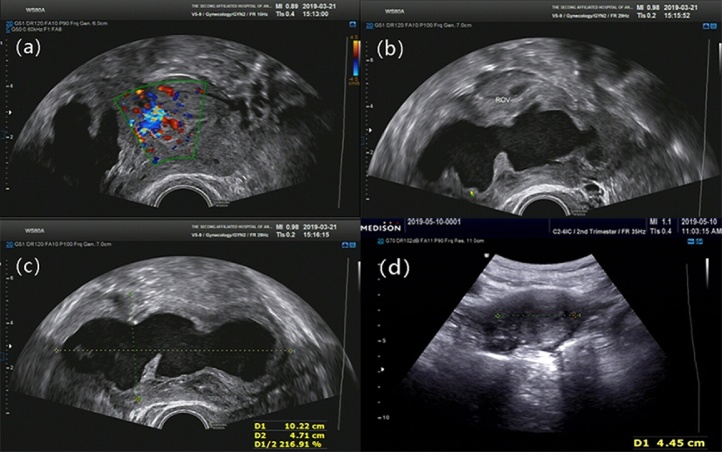
Ultrasound images. (a) Blood flow signals around the cyst. (b) (c) Cyst on the right side of the uterus. (d) One month after surgery.

**Fig. 2:**
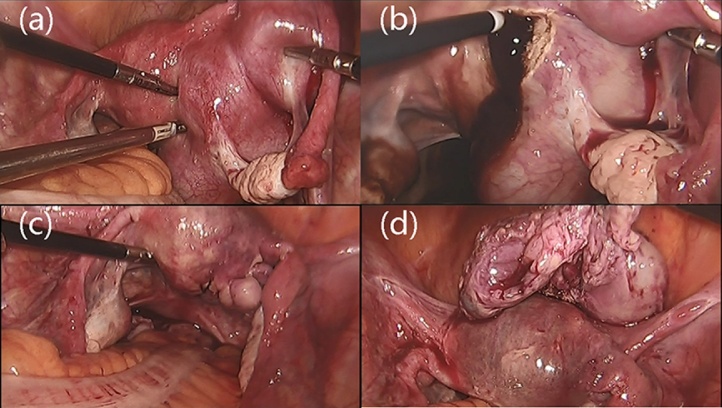
Laparoscopic images. (a) Cyst appearance. (b) The cystic mass wall was opened, and chocolate-colored fluid was visible. (c) The cavity was sutured. (d) Complete removal of the cyst.

**Fig. 3:**
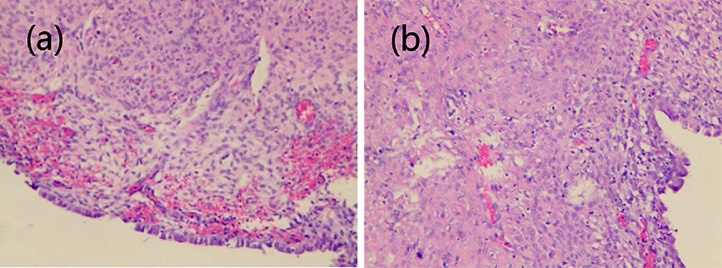
Pathological images (HE 200×).

A 38-year-old, middle-aged woman had a regular menstrual cycle (9/27 days) with normal menses amounts, and her last menstrual period was on 3/9/2019. She had mild dysmenorrhoea, which was relieved within 3 days, without treatment. Starting in December 2018, her dysmenorrhoea worsened. She visited our hospital on 3/22/2019 and received an ultrasound examination, which presented a 104 mm × 55 mm × 60 mm pelvic mass on the right side of the uterus, with dense echoes, and an extremely rich blood signal in the cyst wall (Fig. 1). The patient underwent laparoscopy under general anaesthesia on 3/26/2019. The uterine body tissue was located in the left pelvic cavity. The right side of the uterine protrusion was obvious and bumpy. There were three protrusions connected in a line. The right fallopian tube and round ligament were severely compressed and completely separated, with a distance of 40 mm between them. The right ligament of the right ovary was stretched, while a large amount of tension was maintained on the surface of the uterine protrusion (Fig. 2a). When we opened the posterior wall of the uterus, a large amount of chocolate-colored thick fluid was seen (Fig. 2b). The cystic fluid was fully aspirated, and the cyst was completely removed. The tumour cavity was then sutured with 1-0 V-Loc ™ 180 absorbable and barbed sutures (coviden, CO, USA) (Fig. 2c). The detailed procedure of the laparoscopic surgery is shown in the video (Supplementary materials). Postoperative pathology revealed myometrial cysts and bleeding on the inner wall. Microscopic examination confirmed the presence of endometriotic cysts (Fig. 3). Based on pathology results and laparoscopy findings, cystic adenomyosis was postoperatively diagnosed. Three days after surgery, the patient was discharged. More than one month after surgery, the patient received a repeat pelvic ultrasound. There were multiple strong echogenic spots in the right muscular layer of the uterus, which were considered postoperative changes (Fig. 2). She no longer experienced dysmenorrhoea.

## 3 Discussion

Endometriosis is a common disease in women that is divided into ovarian endometrioid cysts, superficial endometriosis, deep infiltrating endometriosis and other special types according to pathological characteristics. As a special type of endometriosis, the incidence of cystic adenomyosis is low, and its specific incidence is unknown. In 1990, Parulekar [5] reported the first case of cystic adenomyosis, describing the clinical and histopathological characteristics of the disease. Cystic adenomyosis is clinically characterized by the appearance of one or more cystic cavities between the walls of the uterine muscles is the cysts contain chocolate-colored liquid, and the lining of the cystic cavity contains endometrial glands and stroma. The disease is generally asymptomatic in the early stages and can cause dysmenorrhoea and chronic pelvic pain as the disease progresses. Adolescent patients may suffer severe primary dysmenorrhoea [6]. Very large cystic adenomyosis can spontaneously rupture.

Cystic adenomyosis is divided into two types according to the age of onset: adolescent and adult. The most prominent feature in adolescents is the progressive aggravation of dysmenorrhoea after menarche, which suggests that the disease is congenital. The pathogenesis involves Müllerian tube damage or double folding during the development of the residual cystic cavity or residual epithelium after fusion with the contralateral Müllerian tube. After menarche, functional bleeding from the lining of the cystic cavity or epithelium in responses to oestrogen increases cystic cavity pressure, and early progressive dysmenorrhoea occurs [7]. The adult type is common in menopausal women. These women are over 30 years old and have many high-risk factors that cause damage to the endometrium and myometrium, such as a history of abortion, fertility and curettage. When the damage between the endometrium and myometrial junction causes secondary adenomyosis, adenomyosis can develop into cystic adenomyosis. In this case, dysmenorrhoea occurs in patients who had a caesarean section or the induction of labour during menarche. This type of the disease is nonprogressive, with an onset age over 35 years old. This type of the disease is defined as the adult type.

In auxiliary examinations, ultrasound and magnetic resonance imaging (MRI) are helpful for making a timely diagnosis. According to previous reports, ultrasounds suggest the presence of mixed masses and dark areas of cystic fluid. These features often lead clinicians to misdiagnose the disease as uterine fibroid liquefaction, uterine sarcoma, or endometrial lesions. Comparatively speaking, MRI is superior to ultrasound in assisted diagnosis. MRI can not only determine the size and location of the lesion but can also be used to examine its characteristic signals in different weighted images. For example, the lesion had a high signal on the T1-weighted image, a medium to high signal on the T2-weighted image, and a low signal on the periphery of the T2-weighted image. This feature can be used to distinguish this condition from the liquefaction of uterine fibroids [8]. In this case, ultrasound showed a very large pelvic mass, with thick-walled cystic, dense echoes inside, that was 104 mm × 55 mm × 60 mm and located in the right side of the uterus. Gynaecological examination showed a mass of approximately 8 cm in diameter that could be touched in the right appendix area. The first diagnosis was an ovarian cyst. Subsequently, it was confirmed by MRI that this mass was located in the myometrium. Its signals corresponded to the above characteristics, which helped to confirm the diagnosis. The final diagnosis was cystic adenomyosis by pathology.

The laparoscopic resection of lesions has a significant effect on cystic adenomyosis. Nabeshima [9] reported that a 27-year-old patient who was diagnosed with cystic adenomyoma had an unsuccessful laparotomy and severe dysmenorrhoea after the operation. Later, laparoscopic resection was attempted. During the operation, laparoscopic ultrasound was used to detect lesion locations and boundaries. The cyst was completely removed, and the postoperative dysmenorrhoea completely disappeared. The choice of the surgical method for this patient was based on the following three points. 1. The patient was a 38-year-old woman. The preoperative MRI showed that the cyst was located in the myometrium. The surgical field was clear, the patient recovered quickly, and the surgical scar was smaller because laparoscopy was applied. The capsule wall was completely removed during surgery, and the capsule cavity was sutured to avoid recurrence. 2. Dense light spots could be seen in the cyst by ultrasound. Although its volume was large, the contents of the cyst were liquid. During the operation, the cystic fluid was withdrawn, and the cyst volume was reduced to avoid open surgery 3. In this case, as the cyst was very large, if hysteroscopic resection was performed, the cystic cavity would have be too large, and the cyst wall would not have been be completely removed, making it relapse likely. After surgery, the patient was followed up for six months. No dysmenorrhoea occurred, and no recurrence of cysts was found by ultrasound.

## 4 Conclusion

Cystic adenomyosis is rarely reported in the medical literature. Cystic adenomyosis is easy for clinicians to misdiagnose, and its diagnosis can be missed because of its low incidence and rare clinical and atypical clinical features. In this case, a pelvic mass was diagnosed before surgery. During the operation, a large amount of chocolate-colored thick fluid was present in the cyst cavity, which was considered cystic adenomyosis. Laparoscopic surgery is an effective method for the diagnosis and treatment of large cystic masses in the uterine myometrium.

## Declaration of Competing Interest

The authors declare that they have no conflicts of interest.

## Funding

No sources of funding

## Ethical approval

Not required for case reports at our hospital. Single case reports are exempt from ethical approval in our institution.

## Consent

Written informed consent was obtained from the patient for publication of this case report and accompanying images. A copy of the written consent is available for review by the Editor-in-Chief of this journal on request.

## Author contribution

Congqing Li: Managed the patient and did the surgery, design of the study, data interpretation and analysis, revision, wrote the manuscript.

Youjiang Xu: Study design, other.

Lin Cong: Study design, revision, Validation.

## Registration of research studies

NA.

## Guarantor

Congqing Li and Lin Cong.

## Provenance and peer review

Not commissioned, externally peer-reviewed.
